# Budgetary participation and organizational performance in Chinese public hospitals: facilitation or inhibition?

**DOI:** 10.3389/fpubh.2025.1601181

**Published:** 2025-05-19

**Authors:** Qiwen Jiang, Jing Zhou, Xuemei Kuang, Shuang Chen

**Affiliations:** ^1^School of Economics and Management, Southeast University, Nanjing, China; ^2^School of Auditing, Nanjing Audit University Jinshen College, Nanjing, China

**Keywords:** budget participation, organizational performance, public hospital, Latour’s Actor-Network Theory, self-efficacy

## Abstract

**Introduction:**

Against the backdrop of deepening China’s medical and health system reform, public hospitals are responsible for improving modern hospital management systems. This article aimed to understand whether and how public hospital budget participation affects organizational performance in China.

**Methods:**

Based on the novel insights of Latour’s Actor-Network Theory and self-efficiency theory, this article combines qualitative and quantitative research methods. In the qualitative research, this article used Grounded Theory and interviewed ten financial heads of public hospitals. In quantitative research, this article used an empirical research method and distributed 168 questionnaires, of which 164 valid responses were collected for analysis.

**Result and discussion:**

This article reaches the following conclusions: (1) There is a positive correlation between budget participation and non-healthcare performance. (2) At the objective level, there was no significant correlation between budget participation and self-efficacy. From a subjective perspective, budget participation, planning self-efficacy, and interpersonal communication and coordination self-efficacy were significantly and positively correlated. (3) Budget participation could have an effect on NHP through planning self-efficacy, interpersonal communication and coordination self-efficacy The innovations of this article are: firstly, this article reasonably confirms the positive relationship between budget participation and organizational performance of public hospitals in China, providing useful references or subsequent research and other national and regional studies. Second, it analyzes the impact of budget participation on organizational performance based on a new perspective of Latour’s Actor-Network Theory. This article is among the first to apply ANT in the context of hospital budgeting, offering novel theoretical insights. Finally, it uses a combination of qualitative and quantitative research methods to analyze the data.

## 1 Introduction

Budget participation is defined as the practice of allowing subordinates to participate in and influence the budget-setting process (1). Over recent decades, the BP approach has drawn interest among researchers, practitioners, and policymakers (2). In terms of the impact of budgetary participation, existing studies have mainly explored its effects of budgetary participation on organizational performance (3), budgetary slack (4), job satisfaction (5), and self-efficacy (6). However, the existing research on the impact of budgetary participation on organizational performance is controversial (7). One view is that budget participation can promote organizational performance (8). Another view is that budget participation has a negative or no effect on organizational performance (9). This controversy may exist because traditional theories fail to explain the relationship between budgetary participation and organizational performance. In terms of research methodology, most existing studies have used a single quantitative research approach to explore the relationship between budgetary participation and organizational performance (3). Although there have been many studies on the impact of budgetary participation on organizational performance (52), there are fewer studies on the impact of budgetary participation on organizational performance in China, with only six articles (3).

In 2015, the Ministry of Finance’s National Health and Family Planning Commission’s National Bureau of Traditional Chinese Medicine’s Guidance on Strengthening Financial and Budgetary Management of Public Hospitals stated that all public hospitals should establish and implement a comprehensive budget management system by the end of 2016. Thus, the total budget management approach is also widely used in the health care sector (10). Public hospitals have responded to the requirements of the reform of the healthcare system and have implemented comprehensive budget management. The industry and theoretical community have achieved corresponding results in budget management in public hospitals. However, the focus of these results is on the more general issues of the construction of a comprehensive budget management system in public hospitals (10–12). Little research has been conducted on how to improve the overall performance of public hospitals by increasing staff motivation through budgetary participation in the overall budget (49, 51). Nevertheless, research on the impact of budgetary participation on organizational performance is important for improving both operational and budgetary management in public hospitals.

Based on the above analyses, this article attempts to fill the existing research gap. This article introduces Latour’s Actor-Network Theory and self-efficacy theory and uses a combination of qualitative and quantitative research methods. This article investigates the impact of budgetary participation on organizational performance in public hospitals in China and explores the mechanism of self-efficacy to promote the improvement of the level of operational management and budgeting in public hospitals. The innovations of this article are characterized by the following three main points: First, this article confirms the positive relationship between budget participation and organizational performance of public hospitals in China, providing useful references for subsequent research and other national and regional studies. Second, it analyzes the impact of budget participation on organizational performance based on a new perspective of Latour’s Actor-Network Theory. Finally, it uses a combination of qualitative and quantitative research methods to analyze the data.

## 2 Literature review

The main theoretical motivation for this article stems from the increasing number of recent studies that have begun to unify two disparate streams of literature: on one hand, the literature on public organizations and organizational performance and, on the other hand, the literature on budget behavior. Moreover, there is an increasing body of research on accounting practices using agency theory and other traditional theories that may lead to conflicting results. Therefore, a more appropriate and comprehensive method is required for in-depth analyses. In this section, this article offers in sub-sections 2.1, an insight into the positive correlation between the two variables and 2.2, an insight into the negative correlation between the two variables.

### 2.1 The positive correlation between the two variables

The contribution of budget participation to organizational performance can be systematically explained through multi-level theoretical mechanisms and differentiated practice scenarios. From an information integration perspective, the essence of budget participation is to break down information silos within the organization and optimize the quality of decision-making through a two-way flow of data (13). This effect is particularly pronounced in high-uncertainty environments (14). Behavioral science theories further reveal the deeper driving mechanisms of engagement in individuals and teams. The paths of action include the following three: The first is autonomy empowerment. Employees have a greater voice in equipment purchase budgets, and their commitment to production goals increases (15). Second, it enhances competence identity. The self-efficacy of junior managers increases through participation in setting phased budget goals, which directly reduces goal deviation behaviors (2). Third, there is a cross-sectoral synergy. The case of the Government’s Cross-Departmental Budget Working Group demonstrates that collaborative budgeting has led to a higher frequency of information sharing between departments and a reduction in the project delivery cycle (16). Quantitative studies of the effects of weights and measures reveal the law of fit for the budgetary participation model (8). In decentralized organizations, participatory budgeting leads to increased policy responsiveness by shortening the “decision-implementation” chain (17). However, in highly centralized firms, limited participation is more effective than full participation because it avoids decision-making stagnation (18). Digital transformation has amplified the effectiveness of budget participation. Local governments that have deployed blockchain budget tracking systems have seen a significant increase in the frequency of citizen engagement and higher rates of budget execution (19).

### 2.2 The negative correlation between the two variables

While some studies hold the view that budget participation may have a negative effect or no effect on organizational performance. This is mainly because the core stems from a mismatch between the institutional and organizational contexts. In a scenario where strong performance pressures coexist with weak monitoring, budgetary participation may be alienated as a strategic gaming tool (20). Local government department heads create budgetary slack by overestimating costs or underestimating revenues, which directly leads to less efficient resource allocation (21). The mechanism by which this distorted behavior occurs can be divided into three motivating factors. First, under an incentive system that strongly links compensation to budget achievement, managers tend to set aside “safety cushions” to reduce appraisal risk and increase the size of budgetary slack (22). Moreover, when participatory mechanisms lack social accountability, information rent-seeking behavior by management is systematically condoned by the board. Studies have shown that the size of budgetary slack is higher in the unsupervised group than in the supervised group (23). Thirdly, Lack of cultural appropriateness exacerbates formalized budget participation. Adoption of budget proposals by rank-and-file employees is grossly inadequate in countries with high power distances, resulting in participation being reduced to a symbolic process (24). A deeper contradiction is reflected in the disconnect between organizational capacity and the demand for participation (25). The contribution of budgetary engagement to performance plummets in firms where transformational leadership is absent. Managers are unable to translate employee suggestions into actionable programs (3). Weak technical support directly undermines the effectiveness of budget participation. Enterprises with paper-based budget processes experience delayed information integration and versioning confusion. This results in performance gains being offset by the increased time costs associated with budget participation (26).

In summary, existing studies have mainly used goal-setting, agency, and uncertainty theories and other traditional theories to analyze how budget participation influences organizational performance. However, these results are controversial. This may be because existing studies have considered factors within the organization and ignored those outside it. Therefore, this article introduces Latour’s Actor-Network Theory to explain the impact of budgetary participation on organizational performance. More importantly, most existing studies have been conducted using quantitative methods. A single quantitative method may have problems such as scale mismatch and time window mismatch. Therefore, this article attempts to address the shortcomings of previous studies by adopting a combination of directional and quantitative research methods.

## 3 Theoretical framework

### 3.1 The sociology of worth of Latour’s Actor-Network Theory for understanding the relationship between public hospital budget participation and organizational performance

Studies by Dunk and Nouri (27), Shields and Shields (28), and Derfuss (29) that focus on budget participation often only explain a small portion of budget efficiency and organizational performance. One possible reason for this problem is that research on budget participation often focuses on interactions within a binary system. However, budget goal setting spans the entire organization, with different actors involved in budget participation, such as the government, health agency management, healthcare personnel, and other human actors, thus exceeding the scope of the supervisor-subordinate binary (28, 30). Therefore, this article uses Latour’s Actor-Network Theory, which can comprehensively consider various actors and their interrelationships.

It is evident that past theories can no longer explain the relationship between budget participation and organizational performance. Latour’s Actor-Network Theory has attracted our attention. Latour emphasizes that science can only be understood through its practice; therefore, it is necessary to examine science in action, not just the results of science or simply facts (48). From this perspective, Latour developed an analytical method called “Actor Network Theory” (ANT). According to this method, science survives and develops in a network-like construction process, and this network needs to encompass all social resources and human strategies as much as possible. Latour’s basic orientation is that science is a domain in which neither human nor nonhuman factors are given special priority. This can be seen as a radical form of symmetry theory, which sets the symmetry between human and non-human actors, or rather, super symmetry. Latour’s Actor-Network Theory emphasizes the construction process of social networks and believes that social phenomena are formed by the interconnection of different actors. These connections are dynamic and can be changed through continuous interactions and exchanges. Therefore, the participation of each actor is crucial.

First, when this article applies Latour’s Actor-Network Theory to the field of budget accounting in public hospitals, it views accounting budgeting as a network composed of multiple actors, including human actors (such as management, financial departments, doctors, and nurses) and non-human actors (such as documents, reports, and technical equipment). By analyzing the interactions and impacts between these actors, it is possible to gain a deeper understanding of the relationships between the various actors involved in the budget formulation process.

Second, Latour’s Actor-Network Theory emphasizes the need for cooperation among actors in the budgeting process. Governments, health institutions, doctors, and nurses are all related.

Third, applying Latour’s Actor-Network Theory, there may be competition and conflict between different actors. Government departments may want to control the budget size, while health institution management may want to obtain more budget resources to meet the demand for medical services. Doctors and nurses may want to increase budget investment to improve medical facilities and provide better services. This type of competition and conflict must be weighed and negotiated in the actual budget formulation process.

Finally, Latour’s Actor-Network Theory emphasizes the interactions and influences between actors. During the budgeting process, government departments affect the behavior of health institution management and are also subject to feedback and adjustments from other participants. Through the analysis of Latour’s Actor-Network Theory, researchers can reveal the direct and indirect impact paths of institutions on participant behavior and understand how institutional settings shape participant budgeting behavior. The direct impact path is as follows: the system setter establishes norms and requirements for budget preparation. For example, government departments require the management of health institutions to provide specific financial information in budget reports, which directly affects the financial decision-making and reporting behavior of management in budget preparation. Institutions also influence participants’ behavior through other indirect channels. System setters may require transparency and accountability in the budgeting process, which motivates participants to handle budget-related matters more cautiously to avoid responsibility and punishment in the future.

Based on the above, this article innovatively introduces Latour’s Actor-Network Theory and believes that it is reasonable and necessary to study the relationship between budget participation and the organizational performance of public hospitals. Thus, this article formulates the following hypothesis:

*H1*: Budgetary participation promotes organizational performance.

### 3.2 Experience, theory, and mediating variables

Budget participation is an incentive mechanism that can improve organizational performance (50) and budget effectiveness. Personnel involved in the budget who hold a positive attitude and receive a high degree of motivation can produce improved budget effects (31). This is mainly because the cognitive ability and level of motivation of organization members are greatly improved when they participate in budget management. In addition, the impact of budget participation on organizational performance is influenced by cognitive mechanisms. Budgetary participation can have an informational effect. Under the cognitive mechanism, participating in budgeting is a process of sharing and understanding the information. Therefore, in the process of participating in budgeting, more information can be mastered, role ambiguity can be reduced, and a clearer understanding of the work environment and job responsibilities can be obtained. Organizational performance can be improved by fully utilizing and effectively allocating controllable economic resources (32). Macinati et al. (33) studied the relationship between budget participation and organizational performance in the context of professional hybrids in the healthcare industry. The results showed that the relationship between budget participation and organizational performance was not significant, suggesting the existence of potential mediating effects. As mentioned above, previous experience has taught us that research on the relationship between budget participation and public hospital performance often relies on the involvement of intermediary variables.

Based on theoretical foundations and empirical literature, Latour’s Actor-Network Theory emphasizes network construction and the equal importance of the roles of non-human and human actors. Therefore, when this article examines the relationship between budget participation and organizational performance in public hospitals, there should also be a network construction between the two. Combining the interactivity of theory, this article proposes self-efficacy as an intermediary variable to test the relationship between budget participation and public hospital performance.

Self-efficacy was first proposed by Bandura (34). Since its introduction, various fields have conducted numerous studies on this concept. Bandura (35) elaborated on self-efficacy as “an individual’s evaluation and perception of their ability to complete a task.” The so-called self-efficacy refers to the level of confidence an individual has in whether they can complete a task, which is not related to the skills themselves but to the level of confidence they have in whether they can use their skills to achieve the task. Current measurements of self-efficacy are generally in the form of Likert scales (36). The definition of self-efficacy in this article is based on Bandura’s definition and measures the confidence level of members of public hospital organizations in completing work. However, it not only refers to the self-efficacy of specific job responsibilities but also includes planning self-efficacy, interpersonal communication and coordination self-efficacy, information processing self-efficacy, decision-making, and problem-solving self-efficacy. Western scholars propose that self-efficacy comes from four types of experiences: (1) past successful experiences. (2) Imitation or substitution. There is a lot of knowledge and experience that is not obtained through personal practice but through observation and imitation of the behavior of others (37). If the indirect experiential information conveyed by peer behavior is successful, it can promote the improvement of one’s own self-efficacy. (3) Verbal or social persuasion: If others evaluate individuals as capable of performing a certain task, they will put in more effort, and correspondingly, their self-efficacy will improve. (4) The state of physiology and emotions. A stable, positive, and healthy emotional state can promote self-efficacy, whereas anxiety, tension, or fear can easily weaken it. Fatigue and pain can reduce task-related self-efficacy (38). Bandura’s summary of the sources of self-efficacy is overly broad. Therefore, Gist and Mitchell (51) conducted a more detailed analysis of the factors that affect self-efficacy from three levels: controllability, internal and external sources, and plasticity, and proposed a three-dimensional model of self-efficacy. The model proposed by Gist and Mitchell is more refined and specifies the dimensional characteristics of the influencing factors. Regarding self-efficacy and work effectiveness, Judge and Bono (39) examined the relationship between general self-efficacy and work attitudes and found that employees with higher self-efficacy believe that they can complete tasks well, resulting in higher job satisfaction.

Based on the above analysis, this article formulates the following hypothesis:

*H2*: Self-efficacy mediates between budgetary participation and organizational performance.

## 4 Methodology and results

This article combines directed and quantitative research methods. This is because purely quantitative studies may overlook the contextual factors and psychological mechanisms (e.g., dynamics of self-efficacy) of the participants. Qualitative studies have difficulty demonstrating statistical significance among variables. The mixed methods approach compensates for the shortcomings of a single approach through ‘triangulation’ and enhances the rigor of the article.

### 4.1 Qualitative research

#### 4.1.1 Interview method

Qualitative research was conducted using Grounded Theory in interviews. Grounded Theory (GT) is a qualitative research approach that aims to establish theories based on empirical data (52). This article adopts Grounded Theory for the following reasons: First, the relationship between budgetary participation and organizational performance in public hospitals is multidimensional, dynamic, and context-dependent. Grounded Theory emphasizes the distillation of theory from raw data rather than relying on preconceived assumptions, which is suitable for exploring complex phenomena that are not yet fully understood. Second, budget management in public hospitals involves multiple stakeholders (e.g., government, management, healthcare workers, and patients), and their behavioral motivations and interaction logics need to be understood from the subjective perspectives of the participants. Through open coding and axial coding, Grounded Theory can systematically sort out the core concepts (e.g., ‘self-efficacy’ and ‘non-medical performance’) in the interview data and reveal how budgetary participation affects organizational performance through psychological mechanisms. Finally, previous studies are mostly based on traditional theories (e.g., agency theory), leading to conflicting conclusions or insufficient explanatory power. Grounded theory allows researchers to generate new theoretical frameworks from data (e.g., incorporating Latour’s Actor-Network Theory) to provide more contextualized explanations of the relationship between budgetary participation and performance.

The operational procedures of Grounded Theory generally include: (1) generating concepts from data and logging them step by step; (2) continuously comparing data and concepts and systematically asking generative theoretical questions related to concepts; (3) developing theoretical concepts and establishing connections between concepts; (4) theoretical sampling and systematic encoding of data; and (5) constructing theory, striving to obtain the density, variability, and high degree of integration of theoretical concepts. In the first level of coding (Open Coding), researchers require an open mindset, as much as possible, to “suspend” personal “biases” and research community “fixed opinions” and log all data in their own state. To address bias in data analysis, this article used the member check method. Initial analyses were fed back to the participants to confirm that their views were accurately understood and presented. The main task of secondary coding (also known as associative or axial coding) is to discover and establish various connections between conceptual categories to represent the organic relationships between various parts of the data. These connections can be causal, temporal order, or semantic relationships. Consider their language in the context of the time and the social and cultural context in which they are located, and from the following encoding points. To increase the credibility of the data, the results of the coding were further discussed using peer debriefing, in which no new ideas were generated.

Based on the above, this article uses the GT interview method with ten public hospital-related personnel in China as interviewees to study the relationship between budget participation, self-efficacy, and hospital performance. These 10 people were chosen for this article because they were all directors or financial heads of public hospitals. They started from the grassroots and have a better understanding of the business situation, budgeting, and organizational performance of public hospitals. The sample size was established following the principle of theoretical saturation, whereby the information obtained was collated for each number of readers interviewed, and the interviews ended when the 10th personnel was interviewed and no new significant information was provided. Part of the qualitative analysis of the interview content used the Nvivo software. The details are presented in Table 1.

**Table 1:** Layered coding.

Name	Code type	Folder location	List level	List order
Non-healthcare performance	Node	Node	2	13
Plan	Node	Node	2	5
Decision-making and problem-solving	Node	Node	2	9
Objective practice	Node	Node	2	2
Control	Node	Node	2	10
Interpersonal coordination and communication	Node	Node	2	7
Information processing	Node	Node	2	8
Healthcare Performance	Node	Node	2	12
Hospital performance	Node	Node	1	11
Budget participation	Node	Node	1	1
Staff management	Node	Node	2	6

#### 4.1.2 Data collection

As shown in Table 2, this article selected 10 respondents for in-depth interviews. The interviewees included two hospital-level leaders, three department managers, two business department managers, and three staff members from ten public hospitals. The article uses telephone interviews, and the average interview time is 20 min.

**Table 2:** Interviewees.

No	Name	Professional status	Gender	Interview length
1	Tian Hong	Hospital leader	Male	20 min58
2	Ji Hua	Hospital leader	Female	19 min15
3	Shen Hong	Department Manager	Female	18 min18
4	Hu Ying	Department Manager	Female	25 min45
5	Zhong Yuan	Department manager	Male	19 min17
6	Na Xiaohong	Director of technical office	Female	16 min14
7	Zhang Haiming	Director of technical office	Male	17 min36
8	Fan Ruoyun	Staff	Female	18 min26
9	Li Pan	Staff	Male	19 min47
10	Li Jing	Staff	Female	26 min35

#### 4.1.3 Interview topic

According to Latour’s Actor-Network Theory, using the interview method of GT, five interview outlines were designed in the interview outline. Table 3 presents the first-level dimension involved in the interview outline as budget participation, and the second-level dimension as objective practice. The first-level dimension involved in the second interview outline is budget participation, and the second-level dimension is the perspective of self-awareness. The first-level dimension involved in the third interview outline was self-efficacy, while the second-level dimension was self-efficacy planning, self-efficacy employee management, self-efficacy interpersonal coordination and communication, and self-efficacy information processing. The first-level dimension involved in the fourth interview outline was self-efficacy, and the second-level dimension was self-efficacy decision-making and problem-solving, and self-efficacy control. The first-level dimension involved in the fifth interview outline is hospital performance, and the second-level dimension is hospital performance – healthcare performance and hospital performance – non-healthcare performance.

**Table 3:** Interview topic.

Serial number	Interview outline	First dimension	Secondary dimension
1	Do you think the hospital in question can implement a series of budgeting processes based on a combination of top and bottom processes? Is there a clear budget meeting communication system and communication channel between the management structure and budget units? Is there a multi-dimensional data warehouse technology used for budgeting work in information technology, and has interfaces been established with other systems?	Budget participation	Objective practice
2	Have you participated in the formulation of budget goals? If you change the budget goals? Will your superiors explain the reasons? Can you proactively express your opinions and have a certain voice? Have you invested more time and energy in the preparation work? Have you received help and guidance from your superiors?	Budget participation	Self-awareness
3	Can you develop a reasonable work plan for your unit, allocate resources such as personnel, property, and other resources reasonably, and arrange and allocate time reasonably? (Leader interviewees) Can you objectively evaluate the work performance of subordinates? If you find difficulties and negative emotions in subordinates, are you willing to provide help and believe that it can create a good team atmosphere? Can you establish a good and trusting relationship with suppliers? And can you effectively communicate with others when there are disagreements? Can you actively collect information and pass it on to subordinates, providing effective information for decision-makers? Can you have a good understanding of the instructions conveyed by superiors?	Self-efficacy	Self-efficacy – Planning, Self-efficacy – Employee Management, Self-efficacy – Interpersonal Coordination and Communication, Self-efficacy – Information Processing
4	Can you effectively complete the tasks assigned by the unit, make decisive decisions, take timely action to solve crises, timely grasp information related to new tasks and projects, and believe that you can control work progress?	Self-efficacy	Self-efficacy – decision-making and problem-solving; Self-efficacy – Control
5	Do you feel that the medical quality and safety level of your hospital are constantly improving? What is the performance related to it? Has there been an improvement in patients and satisfaction? Is there a growing trend in reputation and market share? How is the cost control of the hospital?	Hospital performance	Hospital performance – healthcare performance; Hospital performance – non-healthcare performance

#### 4.1.4 Qualitative analysis findings

Through interviews with different subjects, this article obtained the relationship between budget participation and organizational performance in public hospitals and verified that the intermediary variable self-efficacy preliminarily determined under Latour’s Actor-Network Theory framework does indeed promote organizational performance under budget participation.

(1)   Public hospitals can establish a comprehensive budget platform by designing comprehensive budget participation processes and systems, establishing clear budget meeting communication systems and channels, conducting appropriate and diverse budget communication, and improving employee and patient satisfaction and the public hospital’s reputation. From a subjective perspective, budget participation in non-healthcare performance is positively correlated. Therefore, public hospitals should strive to increase their staff’s perception of budget participation, thereby improving work efficiency, employee and patient satisfaction, and their reputation.
(2)   Self-efficacy is more biased toward subjective perception of confidence in completing tasks, and there is a cognitive variable between it and budget participation from a practical perspective. From a subjective perspective, there is a significant positive correlation between budget participation and planning self-efficacy, and between interpersonal communication and coordination self-efficacy. Therefore, by promoting the participation of public hospital staff in budget preparation, improvement, and other processes, it is possible to encourage employees to reasonably and effectively allocate time, develop complete work task plans, carry out work according to plans, establish a frank and mutual trust relationship with other members, encounter disagreements in their work, and communicate effectively.
(3)   Budget participation cognition influences non-healthcare performance through planning self-efficacy, interpersonal communication, and coordination self-efficacy.

### 4.2 Quantitative research

Researchers conducted an empirical research design to collect the information and data needed for the empirical study of this article by means of a questionnaire survey, and to refine and derive the corresponding scales with reference to domestic and international scholars’ measures of budget participation, self-efficacy, and organizational performance in public hospitals.

#### 4.2.1 Data collection

The quantitative data of our public hospitals cannot be disclosed to the public, as in the case of enterprises, due to sensitivity issues. Moreover, Budgetary participation, self-efficacy, and organizational performance variables are mostly measured in national and international studies in the form of Likert scales. Thus, this article used questionnaires to collect data. The main steps taken in this article to ensure the validity of the data are as follows: (1) To ensure the validity of the structure and content of the questionnaire, this article first sorted out the measurement items of budgetary participation, self-efficacy, and organizational performance of public hospitals based on domestic and international studies, drew on widely used scales, and improved them. Subsequently, the researchers asked experts in public hospital management to assist in judging the reasonableness of the design of each measurement item. (2) Pre-testing of the questionnaire, inviting people to complete the questionnaire, improving some of the content based on their responses, and estimating the time required to complete the questionnaire. (3) In this article, we designed a web-based questionnaire with reasonable prompts to remind participants of the precautions for completing the questionnaire to reduce non-response bias. (4) Reliability and validity tests were conducted for each variable of the questionnaire.

With the help of the financial leaders of relevant public hospitals, the questionnaires were distributed through seminars on comprehensive asset and price management in public hospitals. The distribution of respondents was random. A total of 168 questionnaires were collected, and invalid questionnaires were excluded from the analysis. Four were answered incorrectly, and the remaining 164 were analyzed using the statistical analysis software SPSS 25.0.

#### 4.2.2 Variables measurement

##### 4.2.2.1 Budget participation

Milani (40) defined participation in budgeting as a continuous variable to measure the level of involvement of organizational members in the process of budgetary activities and measured it in several ways. The budget participation scale designed by Milani is widely used by management accounting scholars. However, its measure is a budget participant’s subjective perception of self-involvement and influence, which is likely to be subject to cognitive bias, leading to highly unstable results in testing the relationship between the two. Thus, this article adopts a methodology for measuring budgetary participation in terms of objectively occurring managerial practice activities that eliminate information asymmetry, combined with a scale designed by Milani to measure budgetary participation. See Table 4 for specific measurements.

**Table 4:** Measurements of budgetary participation.

Measurement dimension	Item	Measurement of variables and related questionnaire items
Budget participation system	BP11	Your institution follows a top-down process for preparing, balancing, questioning and approving budgets
	BP12	There is a clear communication system for budget meetings and channels of communication between your institution’s budget management body and budget units
	BP13	Your hospital has adopted a professional budget software based on a multi-dimensional data warehouse as a budget management platform to set up the budget logic and execute the budget process.
	BP14	Data interfaces have been established between your institution’s budget system and other major financial and business systems to achieve synergy in functionality and integration at the data level
	BP15	Frequent and varied communication between your institution’s budget management bodies and budget units, as well as between budget units, with timely feedback on the status of budget execution and reasons for variances, etc.
Budget engagement awareness	BP21	You participate in the development of all budget targets
	BP22	If your supervisor changes the budget target, he/she will explain the reasons to you
	BP23	You can often take the initiative to express your opinion
	BP24	You have an important say in setting budget targets
	BP25	You do a lot of preparation for budgeting
	BP26	Your supervisor has discussed setting a budget with you on several occasions

##### 4.2.2.2 Self-efficiency

The development of this part of the scale is based on the Self-Efficacy Scale for Managers in Chinese Enterprises constructed by Ling Wen wheel spokes and Fang Liluo (41), which is mainly used to measure managers’ performance in the four areas of ‘Planning’, ‘Interpersonal Coordination and Communication’, ‘Information Processing’, and ‘Decision-making and Problem-solving’. These dimensions were measured on a 7-point Likert scale of ‘completely disagree – completely agree’ (47), as shown in Table 5.

**Table 5:** Measurement of self-efficiency.

Measurement dimension	Item	Measurement of variables and related questionnaire items
Plan	SE11 SE12 SE13	I believe that I can set up a complete task plan for the unit I believe that I can make a reasonable deployment of resources such as people and materials in the unit I believe that I am always able to allocate and organize my time rationally and efficiently.
Interpersonal Communication and Coordination	SE21 SE22 SE23	I am confident that I can build good relationships with suppliers or patients I am confident that I can build open, trusting relationships with the people I work with I am confident that I can communicate effectively when faced with disagreements at work
Information processing	SE31 SE32 SE33	I am confident that I am able to proactively gather all applicable information and pass it on to my subordinates I am confident that I am able to provide effective information to decision makers (or am a decision maker) I am confident that I am able to understand and carry out instructions communicated to me by my superiors
Decision-making and Problem-solving	SE41 SE42	When a crisis event occurs, I am confident that I can take timely action to resolve it I am confident that I can perform well the tasks or jobs assigned to me by my organization

##### 4.2.2.3 Organizational performance

This article focuses on Chinese public hospitals. China’s healthcare system is still in its infancy, and public financial information and data are not readily available. During the field research, it was evident that the interviewees were very cautious about disclosing specific financial data and information, which is understandable. In addition, public hospitals are non-profit organizations with a greater emphasis on social benefits. Therefore, this article focuses on the non-healthcare service performance of public hospitals. Non-healthcare performance (NHP) mainly includes internal and external customer satisfaction, organizational financial and market performance, as well as the image and reputation of public hospitals in the eyes of the public, and the specific measures are shown in Table 6.

**Table 6:** Measurement of non-healthcare performance.

Measurement dimension	Item	Measurement of variables and related questionnaire items
Non-healthcare performance	NHP1	Increased internal and external customer satisfaction over the past 3 years
	NHP2	Increased financial returns and market share over the past 3 years
	NPH3	Increased reputation over the past 3 years
	NPH4	Increased cost control over the past 3 years

##### 4.2.2.4 Control variables

Considering that other factors also affect the organizational performance of public hospitals, they were included in the model as control variables. The level of public hospitals (Level), type of public hospitals (Status), and area where the public hospitals are located (Area) were selected.

The level of the public hospital can be divided into Level I, Level II, and Level III. Status indicates the category of public hospital, which can be divided into general public hospitals and specialized public hospitals. The area indicates the region where the public hospital is located; 0 indicates Northwest China (covering Shaanxi, Gansu, Ningxia, Qinghai, and Xinjiang), 1 indicates Northeast China (covering Liaoning, Jilin, and Heilongjiang), 2 indicates Southwest China (covering Sichuan, Chongqing, Guizhou, Yunnan, and Tibet), 3 indicates Central China (covering Hubei and Hunan), 4 indicates North China (covering Beijing, Tianjin, Hebei, Shandong, Henan, Shanxi, and Nei Menggu), 5 indicates South China (covering Guangdong, Fujian, Guangxi, and Hainan), and 6 indicates East China (covering Shanghai, Jiangsu, Zhejiang, Anhui, and Jiangxi).

In addition, since budgetary participation and self-efficacy are related to the judgment of one’s own situation, based on the suggestions of previous studies (42–44), some control variables related to situational and individual factors that may affect the results were included in the model, including Tenure, Gender, Age, Background, and Education. Tenure was categorized as follows: Faculty Leaders, Functional Department Management, Operational Section Managers, and General Staff were denoted by 1, 2, 3, and 4, respectively; Age (Age) was categorized into three levels: less than 40 years old, between 40 and 50 years old, and more than 50 years old; and educational attainment was categorized into three levels: college, bachelor’s degree, master’s degree, or doctoral degree.

#### 4.2.3 Descriptive statistics

The distribution of the sample is shown in Table 7. The vast majority of public hospitals in the sample public hospitals studied in this paper are public hospitals of level I, accounting for 89.02 percent, while 8.54 percent of public hospitals are at level II, and 2.44 percent are at level III; 71.95 percent of them are general public hospitals, and 28.05 percent are specialist public hospitals; the location of the public hospitals is mainly concentrated in East China, accounting for 69.51 percent, while the distribution in other regions is more even. The age of the sample’s public hospital personnel was mainly concentrated between 40 and 50 years old, accounting for 56.10 per cent, followed by those under 40 years old, accounting for 28.05 per cent, and those over 50 years old, accounting for 15.85 per cent. The education level of the respondents was relatively high, with 65.85 per cent having a bachelor’s degree and 28.05 per cent having a master’s degree or PhD.

**Table 7:** Sample distribution.

Variables	Type	Frequency	Proportions
Level	I	4	2.44%
	II	14	8.54%
	III	146	89.02%
Status	General	118	71.95%
	Specialized	152	28.05%
Area	East	114	69.51%
	South	6	3.66%
	North	22	13.41%
	Central	6	3.66%
	South West	0	0.00%
	North East	4	2.44%
	North West	12	7.32%
Tenures	Faculty Leaders	24	14.63%
	Functional Department Management	86	52.44%
	Operational Section Management	8	4.88%
	General Staff	46	28.05%
Age	40	46	28.05%
	40–50	92	56.10%
	>50	26	15.85%
Genders	Men	52	31.71%
	Women	112	68.29%
Education	College	10	6.10%
	Bachelor’s degree	108	65.85%
	Master’s degree, or doctoral degree.	46	28.05%

#### 4.2.4 Correlation test

The results of the correlation analysis are shown in Table 8. From Table 8, the correlation coefficient between BPa and NHP is 0.368 which is significant at the 1% level. The correlation coefficient between BPb and NHP was 0.454 which was significant at the 1% level. This preliminarily verifies research hypothesis H1. The correlation coefficients between the variables are less than, and the VIF test is performed are less than 2, and the tolerance values are greater than 0.6. This indicates that there is no multicollinearity among the main variables of this article.

**Table 8:** Correlation analysis of key variables.

Variable	NHP	BPa	BPb	Education	Back ground	Age	Gender	Tenure	Level	Status	Area
NHP	1										
Bpa	0.368^**^	1									
BPb	0.454^**^	0.288^**^	1								
Education	0.113	0.148	0.136	1							
Background	−0.092	−0.161	−0.015	−0.361^**^	1						
Age	−0.015	−0.156	0.07	0.021	−0.085	1					
Gender	0.102	0.092	−0.071	−0.038	0.215^*^	−0.016	1				
Tenure	−0.125	0.057	−0.272^*^	−0.217^*^	0.266^*^	−0.518^**^	0.132	1			
Level	0.032	0.104	0.024	0.246^*^	−0.196	−0.069	−0.001	0.005	1		
Statues	−0.052	−0.069	0.007	−0.034	−0.071	−0.156	−0.033	0.064	0.159	1	
Area	−0.042	0.1	−0.01	−0.06	0.058	−0.146	0.09	0.144	0.054	−0.013	1

^*^p < 0.05; ^**^p < 0.01.

#### 4.2.5 Reliability and validity analysis

In this article, the internal consistency coefficient (Cronbach’s alpha value) was used to test the reliability of each variable in the questionnaire. The analysis results showed that the internal consistency coefficient of each variable exceeded 0.7, as shown in Table 9, indicating that the scale had good consistency and stability. Validity can be divided into content and construct validity. The measurement items used in this article were mature scales developed by scholars with good content validity. The standard factor loadings of each measurement index on their respective latent variables were all higher than 0.6, indicating that the scale could accurately measure each variable and had good convergent validity.

**Table 9:** Reliability and validity analysis.

Measurement dimension	Item	Factor loadings	Cronbach’s α	Variance contribution rate
Budget participation system	BP13	0.836	0.869	65.807%
	BP15	0.833		
	BP11	0.833		
	BP12	0.816		
	BP14	0.734		
Budget engagement awareness	BP26	0.860	0.847	57.424%
	BP24	0.823		
	BP23	0.782		
	BP25	0.780		
	BP21	0.691		
	BP22	0.574		
Non-healthcare performance	NHP1	0.884	0.864	71.181%
	NHP2	0.862		
	NHP3	0.862		
	NHP4	0.762		
Plan	SE11	0.835	0.749	57.548%
	SE12	0.831		
	SE13	0.651		
	SE14	0.699		
Interpersonal Communication and Coordination	SE21	0.732	0.733	65.361%
	SE22	0.858		
	SE23	0.830		
Information processing	SE31	0.799	0.877	73.261%
	SE32	0.813		
	SE33	0.607		
	SE34	0.711		
Decision-making and Problem-solving	SE41	0.892	0.749	80.115%
	SE42	0.880		

#### 4.2.6 Regression analysis

##### 4.2.6.1 Budget participation – organizational performance

As shown in Table 10, the regression analysis found that the budget participation system was significantly and positively correlated with NHP, with a correlation coefficient of 0.362. In the regression model, excluding the explanation part (6%) of Model 1, the incremental explanation for the change in the NHP in Model 2 was (Change in *R*^2^) 11.8%. The above test results show that from an objective point of view, the higher the level of budget participation, the higher the NHP. The perception of budgetary participation was found to be significantly positively correlated with NHP, with a correlation coefficient of 0.486, excluding the explanation part of Model 1 (6%). The incremental interpretation of Model 2 for the changes in NHP was (Change in *R*^2^) of 21.2%. The above test results show that from the perspective of self-perception, the higher the level of budget participation, the higher the NHP.

**Table 10:** Regression results of budget participation and non-healthcare performance.

SerialNumber	Variables	Non-healthcare performance		Non-healthcare performance	
		Beta	*t*	Beta	*t*
1	Constant		0.000		0.000
	Level	0.029	0.230	0.029	0.230
	Status	−0.040	−0.342	−0.040	−0.342
	Area	−0.077	−0.657	−0.077	−0.657
	Tenure	−0.136	−0.968	−0.136	−0.968
	Gender	0.155	1.305	0.155	1.305
	Age	−0.125	−0.928	−0.125	−0.928
	Background	−0.073	−0.562	−0.073	−0.562
	Education	0.072	0.551	0.072	0.551
2	Level	0.010	0.081	0.023	0.206
	Status	−0.009	−0.083	−0.044	−0.428
	Area	−0.098	−0.887	−0.092	−0.886
	Tenure	−0.143	−1.085	0.025	0.196
	Gender	0.114	1.009	0.184*	1.746
	Age	−0.067	−0.521	−0.090	−0.755
	Background	−0.007	−0.056	−0.125	−1.086
	Education	0.041	0.337	0.032	0.273
	BPa	0.362***	3.221		
	BPb			0.486***	4.575
	R^2^	0.060	0.178	0.060	0.271
	ΔR^2^		0.118		0.212
	F	0.578	1.733	0.578	2.980
	ΔF		10.376		20.928

*** means *p* < 0.01, ** means *p* < 0.05, * means *p* < 0.1.

##### 4.2.6.2 Budget participation – self-efficacy

After the regression analysis of self-efficacy in the budget participation system, it was found that there was no significant correlation between the two variables; therefore, the results are not shown here. After the regression analysis of budget participation cognition and self-efficacy, it was found that budget participation cognition and planning, and interpersonal communication and coordination self-efficacy were significantly positively correlated, with correlation coefficients of 0.340 and 0.239, respectively. In the regression model of budget participation self-perception and planning self-efficacy, excluding the explanation part of Model 1, the incremental interpretation of the self-efficacy change in Model 2 was (Δ*R*^2^) at 10.4%. In the regression model of budget participation, self-cognition and interpersonal communication and coordination self-efficacy, excluding the explanation part of Model 1, the incremental interpretation of the self-efficacy change in Model 2 was (Change in *R*^2^) 5.1%. The above test results showed that from the perspective of self-perception, the higher the level of budget participation, the higher the self-efficacy in terms of planning, and interpersonal communication and coordination. The details are presented in Table 11.

**Table 11:** Regression results of budget participation cognition and self-efficacy.

Variables	SEa		SEb		SEc		SEd	
	Beta	*t*	Beta	*t*	Beta	*t*	Beta	*t*
BPb	0.340*** (0.003)	3.116	0.239** (0.044)	2.046	0.183 (0.124)	1.558	0.189 (0.101)	1.661
*R* ^2^	0.231		0.114		0.108		0.162	
Δ*R*^2^	0.104		0.051		0.030		0.032	
*F*	2.409		1.034		0.966		1.544	
Δ*F*	9.711		4.186		2.428		2.757	

*** means *p* < 0.01, ** means *p* < 0.05, * means *p* < 0.1.

##### 4.2.6.3 Budget participation, self-efficacy and organizational performance

Table 12 presents the results of the regression analysis, which found that planning, interpersonal communication and coordination, information processing, decision-making and problem-solving self-efficacy and NHP were significantly positively correlated, with correlation coefficients of 0.448, 0.523, 0.442, and 0.401, respectively. The results showed that the higher the self-efficacy of public hospital staff, the higher the NHP of public hospitals.

**Table 12:** The regression results of self-efficacy and organizational performance.

Variables	NHP		
	Beta	*t*	Overall inspection
SEa	0.448*** (0.000)	4.058	*R*^2^ = 0.235, Δ*R*^2^ = 0.175, *F* = 2.453, Δ*F* = 16.468
SEb	0.523*** (0.000)	5.196	*R*^2^ = 0.316, Δ*R*^2^ = 0.256, *F* = 3.698, Δ*F* = 27.003
SEc	0.442*** (0.000)	4.134	*R*^2^ = 0.240, Δ*R*^2^ = 0.180, *F* = 2.526, Δ*F* = 17.087
SEd	0.401*** (0.001)	3.552	*R*^2^ = 0.200, Δ*R*^2^ = 0.140, *F* = 1.998, Δ*F* = 12.620

*** means *p* < 0.01, ** means *p* < 0.05, * means *p* < 0.1.

Planning, self-efficacy, and NHP were significantly correlated, with a correlation coefficient of 0.316. The perception of budget participation and NHP were significantly correlated, but the correlation coefficient (0.378) was smaller than the correlation coefficient without considering the mediation effect (0.486). Therefore, self-efficacy mediates the relationship between perceptions of budget participation and NHP. Interpersonal communication was significantly correlated with coordination self-efficacy and NHP, with a correlation coefficient of 0.436. The perception of budget participation and NHP were significantly correlated, but the correlation coefficient (0.381) was smaller than the correlation coefficient without mediating effects (0.486). Therefore, interpersonal communication and coordination self-efficacy mediate the relationship between the perception of budget participation and NHP. The details are presented in Table 13.

**Table 13:** Regression results of budget participation, self-efficacy and organizational performance.

Variables	NHP		
	Beta	*t*	Overall statistical test
BPb	0.378*** (0.001)	3.512	*R*^2^ = 0.348, Δ*R*^2^ = 0.288, *F* = 3.789, Δ*F* = 15.699
SEa	0.316*** (0.005)	2.887	
BPb	0.381*** (0.000)	3.954	*R*^2^ = 0.439, Δ*R*^2^ = 0.380, *F* = 5.567, Δ*F* = 24.061
SEb	0.436*** (0.000)	4.615	

*** means *p* < 0.01, ** means *p* < 0.05, * means *p* < 0.1.

**Figure 1:**
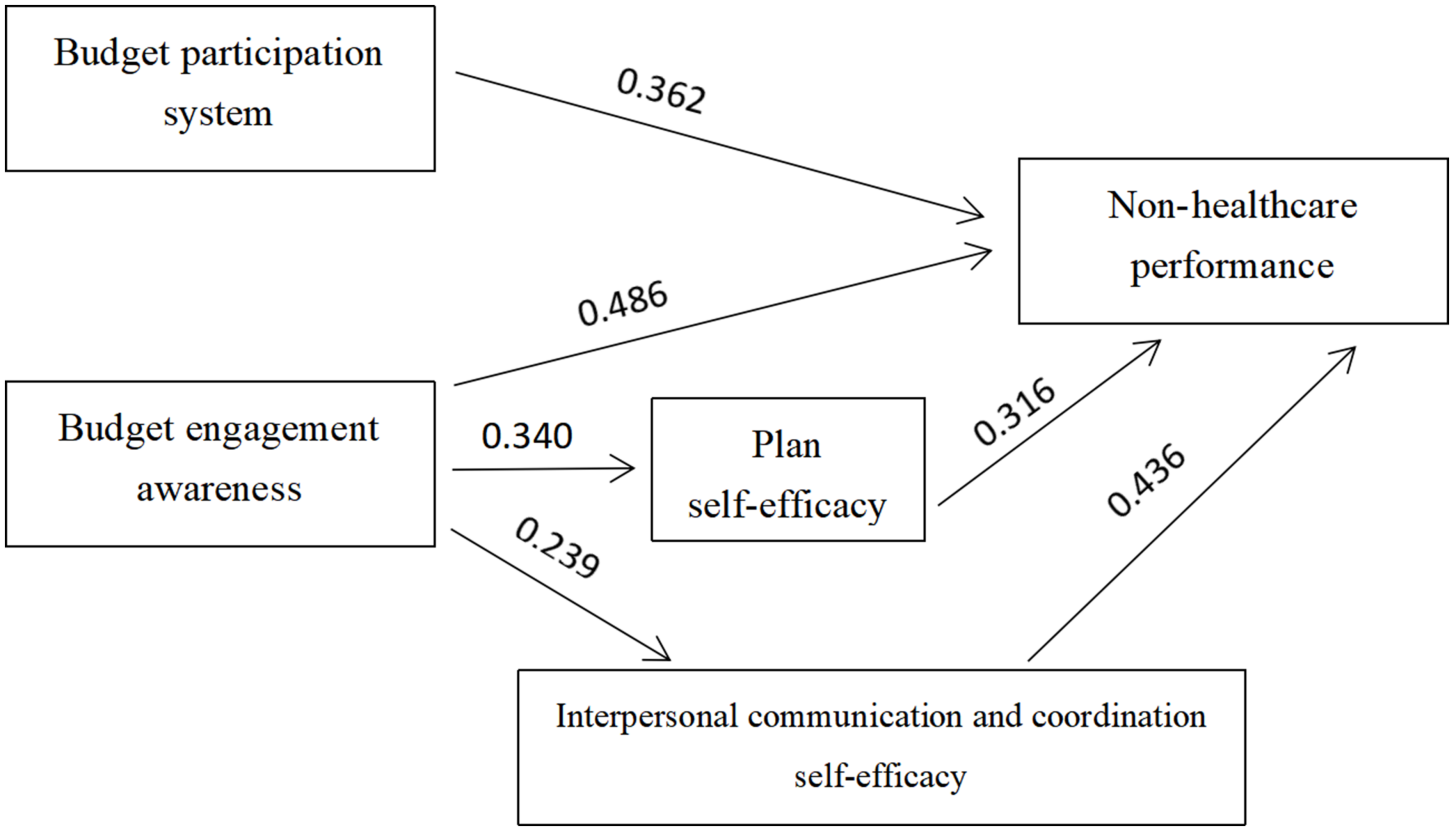
Path analysis of budget participation, self-efficacy and organizational performance in public hospitals.

Figure 1 shows the factor-diameter analysis of the results. The ‘Diameter Diagram’ is essentially a summary of the regression analysis, showing the direct or indirect effects of budget participation (the independent variable) on the organizational performance (the dependent variable) of public hospitals (represented by the factor diameter coefficient, that is, the standard regression coefficients). Thus, this article aims to clarify the causal relationship between the three variables of public hospital budget participation, self-efficacy, and organizational performance.

## 5 Conclusion and discussion

### 5.1 Conclusion

This article obtained 164 valid questionnaires from public hospitals through a questionnaire survey, and the relationship between budget participation and organizational performance in public hospitals was analyzed using a combination of theoretical and empirical methods. Moreover, based on Latour’s Actor-Network Theory, self-efficacy was introduced to examine its role in this. This article found that in the context of public hospitals, budget participation has a significant positive effect on organizational performance, with self-efficacy having a mediating effect. The main conclusions of this article are as follows:

(1)   On an objective and subjective level, budget participation is positively related to the NHP. This further supports existing research that argues that budgetary participation promotes organizational performance (8, 15). Nevertheless, this is contrary to research findings that suggest that budgetary participation has a negative or no effect on organizational performance (20, 22, 24). This may be because this article introduces the new theory of Latour’s actor network and an influx of quantitative research methods. Therefore, public hospitals should build a complete budget platform by designing a sound budget participation process and system, establishing clear communication channels to meet budgets, and conducting appropriate and diverse budget communication to improve employee and patient satisfaction, as well as the public hospital’s reputation. From a subjective analysis perspective, research demonstrates a significant positive correlation between budget participation and NHP outcomes. To leverage this connection, public hospitals should implement the following strategies: First, enhance staff engagement in budgetary processes through training and transparent communication; second, optimize operational efficiency by aligning resource allocation with frontline insights; and third, elevate stakeholder satisfaction through dual-focused improvements in both employee workplace experience and patient care quality. These synergistic enhancements can ultimately strengthen the reputation of institutions within the competitive healthcare landscape.
(2)   At the objective level, there was no significant correlation between budget participation and self-efficacy. These results are inconsistent with those of previous studies (6). This may be because self-efficacy is more biased toward the subjective perception of a person’s degree of confidence in completing a task, and there is an intermediate cognitive variable with budget participation from a practical perspective. From a subjective perspective, budget participation, planning self-efficacy, and interpersonal communication and coordination self-efficacy were significantly and positively correlated. Therefore, by encouraging public hospital staff to participate in the process of budget preparation and improvement, it is possible to motivate staff to allocate their time reasonably and effectively, formulate a complete work task plan, and work according to the plan.
(3)   Planning, interpersonal communication and coordination, information processing, decision-making, and problem-solving self-efficacy were significantly and positively related to NHP. Budget participation can affect NHP through planning self-efficacy, interpersonal communication, and coordination self-efficacy. Therefore, when using budget participation to improve organizational performance, this article suggests paying attention to the role of self-efficacy, especially in the formulation and implementation of plans, effective processing of information, and communication and coordination between employees.

### 5.2 Innovation

The main innovations of this article are as follows.

First, previous studies have conflicting conclusions on budget participation and organizational performance in public hospitals. Some studies suggest a positive relationship between budget participation and organizational performance in public hospitals, while others suggest a negative or no significant effect. In the face of such conflicting situations, this article summarizes and organizes past research viewpoints, and reasonably confirms the positive relationship between budget participation and organizational performance of public hospitals in China, providing useful references for subsequent research and other national and regional studies.

Second, the conflicting views in previous research were largely due to the use of traditional theories that lacked the characteristics of the times. This article innovatively uses Latour’s Actor-Network Theory to identify reasonable mediating variables of self-efficacy. Qualitative methods were used to verify the scientific and rational nature of the model. To explore the relationship between budget behavior and organizational performance accounting, a more effective and comprehensive theoretical path has been found that can be referenced and used by follow-up researchers.

Finally, this article combines qualitative and quantitative analyses to verify the relationship between budget participation, self-efficacy, and organizational performance in public hospitals. Through quantitative analysis of broader survey questionnaire data, this article further verifies that budget participation has a promoting effect on organizational performance.

### 5.3 Limitation

#### 5.3.1 Limitations of the sample

Only 164 valid questionnaires were collected for the quantitative analysis. A total of 69.51 per cent of the hospitals in the sample were concentrated in East China, and the sample was unevenly distributed in other regions, which may have led to geographical bias. Future studies could expand the sample size and cover more regions (e.g., less developed regions in the central and western parts of the country) to enhance the generalizability of the findings.

#### 5.3.2 Methodological limitations of the article

This article uses cross-sectional data and cannot verify the long-term causal relationship between budgetary participation and organizational performance. Self-reported data relying on questionnaires may lead to measurement errors due to social desirability bias or subjective cognitive differences.

Future research could use longitudinal tracking studies to observe the dynamic impact of budgetary participation on organizational performance over time. Without violating academic ethics and morals, future studies could combine objective data (e.g., hospital financial statements, patient visit records) with subjective data to reduce self-reporting bias.

#### 5.3.3 Limitations of theoretical applications

Latour’s Actor-Network Theory emphasizes the reciprocal status of human and non-human actors. However, it was not possible to analyze the specific mechanisms of the role of non-human actors (e.g., budget software and policy documents) in budgetary participation in this article. Future research could further explore the impact of co-communication between human and non-human actors on budgetary participation in the public sector.

## Data Availability

The raw data supporting the conclusions of this article will be made available by the authors, without undue reservation.
