# Supplementary material for: Budgetary participation and organizational performance in Chinese public hospitals: facilitation or inhibition?
Source: Front Public Health. 2025 May 19;13:1601181. doi: 10.3389/fpubh.2025.1601181 (PMC12127427; doi:10.3389/fpubh.2025.1601181)
Supplement: Supplementary file 1 [file Data_Sheet_1.docx]

**Appendix**

**Questionnaire on the Relationship between Budget Participation, Self-efficacy, and Organizational Performance in Public Hospitals**

Dear Sir/Madam:

Hello!

Thank you very much for taking the time out of your busy schedule to support this survey. This survey was conducted on the budget management of public hospitals. The survey was conducted anonymously. We solemnly promise that all information is for academic research and overall analysis purposes only and is strictly confidential. Any individual information about public hospitals will not be disclosed to third parties without written permission. Please read each question carefully and answer based on your actual viewpoint.

Thank you for your support and assistance.

Finally, I wish you good health and a smooth work experience!

Part 1 Basic Information of Public Hospitals and Individuals

1. The level of your public hospital, please fill in: Class ___ Grade ___

2. Type of your hospital: □ Comprehensive □ Specialized

3. The location of your institution, please fill in: ___ Province/ ___ City

4. Your position: □ Hospital level leader □ Functional department manager □ Technical department chief □ General staff member

5. Your department, please fill in:

6. Your gender: □ Male □ Female

7. Your age: □ Under 40 years old □ Between 40 and 50 years old □ Over 50 years old

8. Do you have a management background: □ Yes □ No

9. You have been in this position for ___ years and have been in your institution for ___ years.

10. Your educational background: □ Technical secondary school or below □ Associate degree □ Bachelor’s degree □ Master’s or doctoral degree

Part 2 Budget Participation

Budget management is a complex management system. In order to comprehensively and accurately evaluate the maturity level of budget management in your institution, as well as your personal budget participation in your institution, please choose the corresponding options based on the actual situation and your personal perception.

1. From an objective practical perspective (1=not achieved, 2=partially achieved, 3=basically achieved, 4=fully achieved)

BP11. Your institution conducts budget preparation, balancing, questioning, and approval through a combination of upper and lower processes:

(Not implemented) 1 2 3 4 (fully implemented)

BP12.There is a precise budget meeting communication system and channels between your budget management organization and budget units:

(Not implemented) 1 2 3 4 (fully implemented)

BP13.Your hospital uses professional budget software based on a multidimensional data warehouse as the budget management platform, sets budget logic, and executes budget processes:

(Not implemented) 1 2 3 4 (fully implemented)

BP14.Your budget system and other major financial and business systems have established data interfaces, achieving functional collaboration and data integration:

(Not implemented) 1 2 3 4 (fully implemented)

BP15. Frequent and diverse communication between your budget management organization and budget units, as well as between budget units, timely feedback on budget execution status and reasons for differences:

(Not implemented) 1 2 3 4 (fully implemented)

1. From the perspective of self-awareness (1=completely disagree, 4=basically agree, 7=completely agree, and other scores are given as appropriate)

BP21. You participate in the formulation of all budget goals:

(completely disagree) 1 2 3 4 5 6 7 (completely agree)

BP22. If the superior changes the budget target, they will explain the reason to you:

(completely disagree) 1 2 3 4 5 6 7 (completely agree)

BP23. You can often proactively express your opinions:

(completely disagree) 1 2 3 4 5 6 7 (completely agree)

BP24. You have an important say in setting budget goals:

(completely disagree) 1 2 3 4 5 6 7 (completely agree)

BP25. You have done a lot of budget preparation work:

(completely disagree) 1 2 3 4 5 6 7 (completely agree)

BP26. Your supervisor has discussed with you multiple times to develop a budget:

(completely disagree) 1 2 3 4 5 6 7 (completely agree)

Part 3 Self efficacy

The test items in this section are an explanation of your confidence level in completing work or tasks. Please make judgments and choices based on your actual situation. It is important to note that each test item assumes that you have the authority to complete the task or work. If you are asked to complete the task or work, do you have the confidence to do it well. (1=completely disagree, 4=average, 7=completely agree, and other scores are given as appropriate)

SE11 (Plan). I believe I can set up a complete work plan for my unit.

(completely disagree) 1 2 3 4 5 6 7 (completely agree)

SE12 (Plan). I believe I can make reasonable adjustments to the human, financial, and other resources of my unit.

(completely disagree) 1 2 3 4 5 6 7 (completely agree)

SE13 (Plan). I believe that I can always allocate and arrange my time reasonably and effectively.

(completely disagree) 1 2 3 4 5 6 7 (completely agree)

SE21 (Employee Management). I am confident in objectively and accurately evaluating the work performance of my subordinates.

(completely disagree) 1 2 3 4 5 6 7 (completely agree)

SE22 (Employee Management). I am confident that I can timely identify the difficulties and negative emotions of subordinates and provide assistance.

(completely disagree) 1 2 3 4 5 6 7 (completely agree)

SE23 (Employee Management). I believe I can create a good team atmosphere for the team I lead.

(completely disagree) 1 2 3 4 5 6 7 (completely agree)

SE31 (interpersonal coordination and communication). I am confident in establishing good relationships with suppliers or patients.

(completely disagree) 1 2 3 4 5 6 7 (completely agree)

SE32 (interpersonal coordination and communication). I am confident that I can establish a frank and mutual trust relationship with employees I work with.

(completely disagree) 1 2 3 4 5 6 7 (completely agree)

SE33 (Interpersonal Coordination and Communication). When encountering disagreements at work, I believe I can effectively communicate with others.

(completely disagree) 1 2 3 4 5 6 7 (completely agree)

SE41 (Information Processing). I believe I can proactively collect various applicable information and pass it on to subordinates.

(completely disagree) 1 2 3 4 5 6 7 (completely agree)

SE42 (Information Processing). I believe I can provide effective information for decision-makers (or myself as decision-makers).

(completely disagree) 1 2 3 4 5 6 7 (completely agree)

SE43 (Information Processing). I am confident that I can understand and implement the instructions conveyed by superiors well.

(completely disagree) 1 2 3 4 5 6 7 (completely agree)

SE51 (Decision-making and Problem-solving). When a crisis occurs, I believe I can take timely action to solve it.

(completely disagree) 1 2 3 4 5 6 7 (completely agree)

SE52 (Decision-making and Problem-solving). I am confident that I can complete the tasks or tasks assigned to me by the unit well.

(completely disagree) 1 2 3 4 5 6 7 (completely agree)

SE53 (Decision-making and Problem-solving). I believe I can make decisions correctly and decisively when necessary.

(completely disagree) 1 2 3 4 5 6 7 (completely agree)

SE61 (control). I am confident that I can timely grasp new projects or information related to tasks.

(completely disagree) 1 2 3 4 5 6 7 (completely agree)

SE62 (Control). I believe I can control the progress of the work and make it proceed according to the originally set plan.

(completely disagree) 1 2 3 4 5 6 7 (completely agree)

SE63 (Control).I believe I can effectively supervise and control the work of my unit.

(completely disagree) 1 2 3 4 5 6 7 (completely agree)

Part 4 Performance of Public Hospitals

The various measurement items in this section are about the performance of your institution. Please comprehensively consider your institution’s performance in the past 1-3 years and make judgments and choices on the statements of these measurement items. (1=completely disagree, 4=basically agree, 7=completely agree, and other scores are given as appropriate)

HP1 (Healthcare Performance): Over the past three years, your hospital’s medical quality and safety level have been continuously improving.

(completely disagree) 1 2 3 4 5 6 7 (completely agree)

HP2 (Healthcare Performance): Over the past three years, the average length of stay in your hospital has been decreasing year by year.

(completely disagree) 1 2 3 4 5 6 7 (completely agree)

HP3 (Healthcare Performance): Over the past three years, the number of bed turnover in your hospital has been decreasing year by year.

(completely disagree) 1 2 3 4 5 6 7 (completely agree)

HP4 (Healthcare Performance): Over the past three years, the daily average number of doctors in your hospital has been continuously increasing.

(completely disagree) 1 2 3 4 5 6 7 (completely agree)

HP5 (Healthcare Performance): Over the past three years, your hospital’s physicians have been continuously increasing their daily burden of inpatient bed days.

(completely disagree) 1 2 3 4 5 6 7 (completely agree)

NHP1 (Non-healthcare Performance): Over the past three years, the satisfaction of patients and employees in your hospital has been continuously improving.

(completely disagree) 1 2 3 4 5 6 7 (completely agree)

NHP2 (Non-healthcare Performance): Over the past three years, your hospital’s financial revenue and market share have continued to grow.

(completely disagree) 1 2 3 4 5 6 7 (completely agree)

NHP3 (Non-healthcare performance), your hospital’s reputation has been continuously improved in the past three years.

(completely disagree) 1 2 3 4 5 6 7 (completely agree)

NHP4 (Non-healthcare Performance): Over the past three years, your hospital’s cost control effectiveness has been improved.

(completely disagree) 1 2 3 4 5 6 7 (completely agree)
